# Increased Risk of Stroke after Septicaemia: A Population-Based Longitudinal Study in Taiwan

**DOI:** 10.1371/journal.pone.0089386

**Published:** 2014-02-21

**Authors:** Jiunn-Tay Lee, Wen Ting Chung, Jin-Ding Lin, Giia-Sheun Peng, Chih-Hsin Muo, Che-Chen Lin, Chi-Pang Wen, I-Kuan Wang, Chun-Hung Tseng, Chia-Hung Kao, Chung Y. Hsu

**Affiliations:** 1 Department of Neurology, Tri-Service General Hospital, National Defense Medical Center, Taipei, Taiwan, Republic of China; 2 Graduate Institute of Clinical Medicine, Taipei Medical University, Taipei, Taiwan, Republic of China; 3 School of Public Health, National Defense Medical Center, Taipei, Taiwan, Republic of China; 4 Department of Public Health, China Medical University Hospital, Taichung, Taiwan, Republic of China; 5 Management Office for Health Data, China Medical University Hospital, Taichung, Taiwan, Republic of China; 6 Institute of Population Health Sciences, National Health Research Institutes, Zhunan, Taiwan, Republic of China; 7 Graduate Institute of Clinical Medical Science, China Medical University, Taichung, Taiwan, Republic of China; 8 Department of Internal Medicine, College of Medicine, China Medical University, Taichung, Taiwan, Republic of China; 9 Division of Kidney Disease, China Medical University Hospital, Taichung, Taiwan, Republic of China; 10 Department of Neurology, China Medical University Hospital, Taichung, Taiwan, Republic of China; 11 Graduate Institute of Clinical Medicine Science and School of Medicine, College of Medicine, China Medical University, Taichung, Taiwan, Republic of China; 12 Department of Nuclear Medicine and PET Center, China Medical University Hospital, Taichung, Taiwan, Republic of China; Murdoch Childrens Research Institute, Australia

## Abstract

Inflammation and infection have been noted to increase stroke risk. However, the association between septicaemia and increased risk of stroke remains unclear. This population-based cohort study, using a National Health Insurance database, aimed to investigate whether patients with septicaemia are predisposed to increased stroke risk. The study included all patients hospitalised for septicaemia for the first time between 2000 and 2003 without prior stroke. Patients were followed until the end of 2010 to evaluate incidence of stroke. An age-, gender- and co-morbidities-matched cohort without prior stroke served as the control. Cox’s proportional hazards regressions were used to assess differences in stroke risk between groups. Based on hazard ratios (HRs), patients with septicaemia had greater stroke risk, especially in the younger age groups (age <45: HR = 4.16, 95% CI: 2.39–7.24, *p*<0.001; age 45–64: HR = 1.76, 95% CI: 1.41–2.19, *p*<0.001; age ≥65: HR = 1.05, 95% CI: 0.91–1.22, *p*>0.05). Haemorrhagic stroke was the dominant type (ischaemic stroke: HR = 1.20, 95% CI: 1.06–1.37, *p*<0.01; haemorrhagic stroke: HR = 1.82, 95% CI: 1.35–2.46, *p*<0.001) and patients without co-morbidities were at slightly higher risk (without co-morbidities: HR = 1.49, 95% CI: 1.02–2.17, *p*<0.05; with co-morbidities: HR = 1.24, 95% CI: 1.10–1.41, *p*<0.001). The impact of septicaemia on stroke risk was highest within 6 months of the event and gradually declined over time. Our results suggest that septicaemia is associated with an increase in stroke risk, which is greatest in haemorrhagic stroke. Closer attention to patients with history of septicaemia may be warranted for stroke preventive measures, especially for younger patients without co-morbidities.

## Introduction

Stroke is an important cause of death and disability worldwide. Improving stroke prevention is more cost-effective than developing effective stroke therapy. In Taiwan, stroke is the leading cause of adult disability, the third leading cause of death, and the second highest healthcare expenditure for adults aged 65 and older [1], [2]. Analysis of the national stroke profile and data maintained by the Taiwan Stroke Registry [2] shows that the Get with the Guidelines (GWTG) Program of the American Heart Association and American Stroke Association [3], [4] is applicable in Taiwan. In addition to setting performance standards, the GWTG Program aims to improve stroke prevention and reduce healthcare expenditures by promoting public and professional awareness of risk factors for stroke [3], [4]. The major risk factors for stroke have been established, and identification of these can help in the development of preventive measures. However, up to 40% of stroke patients may have health conditions other than the established stroke risk factors, that predispose a selected population to stroke [5], [6]. Exploration of selected health conditions to develop a more comprehensive list of stroke risk factors may improve stroke prevention.

Acute infection may have a short-term impact as a trigger of stroke [7]–[12]. However, the causal role of inflammation/infection in stroke pathogenesis has not been firmly established [13]. Although evidence points to acute and chronic infections acting in combination with conventional risk factors to increase risk of stroke, it has not yet been determined whether an acute infection such as septicaemia (acute and serious systemic infection) could independently increase risk of stroke in the absence of established stroke risk factors. In addition to classic risk factors for stroke, it is important to explore the impact of acute inflammation and infection, especially severe episodes such as septicaemia, on stroke risk, since there is currently no definitive association reported between acute infection and stroke except for a short-term increase in stroke risk [7], [8].

The availability of a large database from the Taiwan National Health Insurance program which represents 98% of the population in Taiwan provided an opportunity for us to investigate the relationship between hospitalisation for septicaemia and subsequent stroke. The purpose of this study was to explore whether a history of septicaemia is accompanied by a subsequent increase in the risk for developing stroke.

## Methods

### Database

This study used the Longitudinal Health Insurance Database (LHID) released by the Taiwan National Health Research Institute in 2011. LHID contains data from original claims of one million beneficiaries, randomly sampled from the registry for the Beneficiaries of the National Health Insurance Research Database (NHIRD) where more than 23 million individuals are enrolled into the National Health Insurance (NHI) program, the universal payer for healthcare in Taiwan [14]. The LHID contains records on inpatient, outpatient and ambulatory care services, covering the period from 1996 to 2010. The disease diagnosis accepted by the NHI is the International Classification of Diseases, Ninth Revision, Clinical Modification (ICD-9-CM). NHI has a rigorous monitoring system to ensure that the claims for reimbursement for healthcare are based on valid diagnoses. NHI randomly audits the healthcare records to validate medical claims for diagnosis and treatment. Previous reports have shown the reliability of the diagnosis coding in the LHID [15], [16]. This epidemiological research project was approved by China Medical University Institute Review Board (CMU-REC-101-012). Because data in the LHID is de-identified, the signed informed consent of participants was waived.

### Study Samples

The study group and a control cohort were selected from the LHID. The diagnoses of septicaemia and stroke were the basis for claims for reimbursement for relevant services rendered by the hospitals and physicians. NHI maintains stringent regulations and periodically audits the claims field for reimbursement. The study group comprised all patients who had been hospitalised for septicaemia based on ICD-9-CM codes 003.1, 036.2 and 038 for the first time from 2000 to 2003 (N = 7,611). Patients admitted for treatment of septicaemia before 2000 were excluded to increase the likelihood of identifying new cases. We also excluded patients with diagnosis of stroke (ICD-9-CM codes 430 to 438) before the diagnosis of septicaemia (N = 1,288). Patients who were hospitalised with septicaemia and who died during hospitalisation or within 7 days after discharge (N = 1,695) were also excluded. We excluded patients who died within 7 days of discharge, since based on Taiwanese customs, a large percentage of Taiwanese patients prefer to die at home, rather than in a hospital. We therefore presumed that patients who died within 7 days of discharge were in the same category as patients who died during hospitalisation. The final study group contained 4,628 cases. The first hospitalisation from the beginning of 2000 to the end of 2003 during which a patient was diagnosed with septicaemia, was set as the index date. We randomly selected 18,399 subjects (a sample size 4-fold that of the septicaemia group), frequency matched with the study cohort in terms of age, gender and co-morbidities including hypertension, diabetes, ischaemic heart diseases, atrial fibrillation and dyslipidaemia and index date. Each patient was then followed up from the index date until the occurrence of stroke. For those who did not have stroke, the last day of follow-up was defined as the date of insurance withdrawal or the last day of the study period (December 31, 2010).

### Definitions of Stroke Subtypes by ICD Classification

Stroke subtypes were classified into haemorrhagic stroke (ICD-9-CM codes 430–432) and ischaemic stroke (ICD-9-CM codes 433–437). Computed tomography (CT) or magnetic resonance imaging (MRI) was used to further confirm the diagnosis of stroke and to confirm that the patient was a new stroke case rather than somebody with a history of stroke. If the patient only had ICD codes of stroke but no procedure coding of CT or MRI, the diagnosis of stroke was not made. Co-morbidities that are also stroke risk factors were established before the index date based on outpatient and inpatient data with the following ICD codes: hypertension (ICD-9-CM codes 401–405), diabetes (ICD-9-CM code 250), ischaemic heart diseases (ICD-9-CM codes 410–414), dyslipidaemia (ICD-9-CM code 272) and atrial fibrillation (ICD-9-CM code 427.3).

### Statistical Analysis

Continuous variables were presented as mean ± SD and categorical variables as frequencies and percentages. Differences between study group and control cohort in the distribution of demographic characteristics (age and gender) and co-morbidities (hypertension, diabetes, ischaemic heart diseases, dyslipidaemia and atrial fibrillation) were examined by Pearson chi-square test. Cox’s proportional hazard regression analysis was performed to calculate crude and adjusted hazard ratios (HR), with 95% confidence intervals (CI), for the impact of septicaemia on developing stroke. To investigate the interaction of covariates in relation to the association of septicaemia and stroke, we also calculated crude and adjusted HR stratified by age (<45, 45 to 64, ≥65 years), gender, co-morbidities, and follow-up time. We also tested the relationship between Schoenfeld residuals for septicaemia and follow-up time in the Cox’s proportional hazard model for assessing the risk of stroke. All statistical analyses were performed with SAS software version 9.2 (SAS Institute Inc., Cary, NC). A two-tailed *p* value less than 0.05 was considered statistically significant.

## Results

Baseline characteristics of the study group and the control cohort are shown in Table 1. There were no significant differences in distribution of age, gender, and co-morbidities between the two groups. There were no significant differences in baseline characteristics between the study group with septicaemia and the control cohort. Over the course of a 7–10 year follow-up period, there were 335 (incidence: 16.06/1,000 person-years) and 1,593 (incidence: 14.32/1,000 person-years) stroke events in the study group and the control cohort, respectively (Table 2). In the subgroup analysis, patients with septicaemia had a consistently higher cumulative incidence of stroke compared to those in the control cohort. The age-adjusted HR for developing stroke of any type among patients with septicaemia was 1.27 (95% CI: 1.13–1.43, *p*<0.001). The age-adjusted HRs were 1.20 (95% CI: 1.06–1.37, *p*<0.01) and 1.82 (95% CI: 1.35–2.46, *p*<0.001) for developing ischaemic and haemorrhagic stroke, respectively.

**Table 1:** Baseline characteristics of the septicaemia group and the control cohort.

	Patients with Septicaemia† (N = 4,628)		(N = 18,399)		Control Cohort
Variables	n	%	n	%	*p*-value
Age, years					0.96
<45	1344	29	5362	29.1	
45–64	1215	26.3	4853	26.4	
≥65	2069	44.7	8184	44.5	
Mean ± SD	55.4	−25.2	55.1	−24.9	0.54
Gender					0.93
Male	2483	53.6	9885	53.7	
Female	2145	46.4	8514	46.3	
Co-morbidities					
Hypertension	2193	47.4	8718	47.4	>0.99
Diabetes	1472	31.8	5819	31.6	0.81
Ischaemic Heart Diseases	1343	29	5310	28.9	0.83
Atrial Fibrillation	215	4.65	780	4.24	0.22

† ICD9 003.1, 036.2 & 038.

Chi-square test.

**Table 2:** Hazard ratios for development of stroke associated with septicaemia.

	Patients with Septicaemia				Control Cohort
Variable	No. of events	Incidence, per 1,000person-years	No. of events	Incidence, per1,000 person-years	Age-adjusted HR (95% CI)
Overall					
All Strokes	335	16.06	1593	14.32	1.27 (1.13–1.43)***
Ischaemic Stroke	278	13.53	1425	12.81	1.20 (1.06–1.37)**
Haemorrhagic Stroke	57	2.77	184	1.65	1.82 (1.35–2.46)***
With co-morbidities					
All Strokes	296	27.51	1452	23.12	1.24 (1.10–1.41)***
Ischaemic Stroke	257	23.89	1314	20.92	1.19 (1.05–1.37)**
Haemorrhagic Stroke	44	4.09	152	2.42	1.71 (1.22–2.40)**
Without co-morbidities					
All Strokes	34	3.47	141	2.91	1.49 (1.02–2.17)*
Ischaemic Stroke	21	2.14	111	2.29	1.20 (0.75–1.91)
Haemorrhagic Stroke	13	1.33	32	0.66	2.32 (1.21–4.43)*

HR: hazard ratio, CI: confidence interval.

*p<0.05; **p<0.01; ***p<0.001.

The age-adjusted HR for stroke of any type was higher in the septicaemia group with co-morbidities as compared to the control cohort with co-morbidities (HR = 1.24, 95% CI: 1.10–1.41, *p*<0.001), and patients in the co-morbidities group with ischaemic stroke had an HR of 1.19 (95% CI: 1.05–1.37, *p*<0.01) compared to patients with haemorrhagic stroke (HR = 1.71, 95% CI: 1.22–2.40, *p*<0.01) (Table 2). The age-adjusted HR for stroke of any type was also higher in the septicaemia group without co-morbidities as compared to the control cohort without co-morbidities (HR = 1.49, 95% CI: 1.02–2.17, *p*<0.05), and patients without co-morbidities who had ischaemic stroke had an HR of 1.20 (95% CI: 0.75–1.91, *p*>0.05) compared to patients with haemorrhagic stroke (HR = 2.32, 95% CI: 1.21–4.43, *p*<0.05) (Table 2). The impact reflected by HRs appears slightly greater among those without co-morbidities. However, because of smaller case numbers, the confidence intervals were wider in the groups without co-morbidities (Table 2).

Table 3 presents results of further analysis of patient data stratified by age (<45 years; 45–64 years; ≥65 years) and gender. The risk for developing stroke was inversely associated with age (interaction *p*<0.05). The HR for developing stroke of any type was significantly higher in the septicaemia group than in the control cohort in the <45 years age group (HR = 4.16, 95% CI: 2.39–7.24, *p*<0.001). Patients in this group with ischaemic stroke had a significantly lower HR (2.08, 95% CI: 1.02–4.25, *p*<0.05), compared to those with haemorrhagic stroke (HR = 28.6, 95% CI: 6.46–127, *p*<0.001). The impact of septicaemia on stroke risk declined in the 45–64 years age group (stroke of any type, HR = 1.76, 95% CI: 1.41–2.19, *p*<0.001; ischaemic stroke, HR = 1.77, 95% CI: 1.39–2.25, *p*<0.001; haemorrhagic stroke, HR = 1.68, 95% CI: 0.98–2.90, *p*>0.05), and shows no significant difference between the septicaemia group and the control cohort with age ≥65 years (stroke of any type, HR = 1.05, 95% CI: 0.91–1.22, *p*>0.05; ischaemic stroke, HR = 1.03, 95% CI: 0.88–1.20, *p*>0.05; haemorrhagic stroke, HR = 1.34, 95% CI: 0.88–2.04, *p*>0.05). The interaction *p* value between age and septicaemia was <0.05 for all types of stroke, ischaemic and haemorrhagic stroke. The interaction *p* value between gender and septicaemia was 0.042 for haemorrhagic stroke, but >0.05 for all types of stroke and ischaemic stroke. In addition, the adjusted HR for developing haemorrhagic stroke in patients with septicaemia compared with controls was 1.10 (95% CI: 0.65–1.87) among females (*p*>0.05) and 2.08 among males (95% CI: 1.45–3.00, *p*<0.001).

**Table 3:** Hazard ratios for the development of stroke associated with septicaemia by age and gender.

	Patients with Septicaemia				Control Cohort
Variable	No. of events	Incidence, per 1,000person-years	No. of events	Incidence, per1,000 person-years	HR (95% CI)
Age, years					
<45					
All Strokes	24	2.87	26	0.69	4.16 (2.39–7.24)***
Ischaemic Stroke	11	1.32	24	0.63	2.08 (1.02–4.25)*
Haemorrhagic Stroke	13	1.56	2	0.05	28.6 (6.46–127)***
45–64					
All Strokes	105	18.45	330	10.54	1.76 (1.41–2.19)***
Ischaemic Stroke	89	15.64	279	8.91	1.77 (1.39–2.25)***
Haemorrhagic Stroke	17	2.99	55	1.76	1.68 (0.98–2.90)
≥65					
All Strokes	201	30.92	1237	29.43	1.05 (0.91–1.22)
Ischaemic Stroke	178	27.39	1122	26.69	1.03 (0.88–1.20)
Haemorrhagic Stroke	27	4.15	127	3.02	1.34 (0.88–2.04)
Gender					
Male					
All Strokes	171	15.89	841	13.99	1.13 (0.95–1.33)
Ischaemic Stroke	744	12.38	134	12.45	1.00 (0.83–1.20)
Haemorrhagic Stroke	40	3.72	105	1.75	2.08 (1.45–3.00)***
Female					
All Strokes	159	16.24	752	14.71	1.10 (0.95–1.30)
Ischaemic Stroke	144	14.71	744	12.38	1.10 (0.92–1.31)
Haemorrhagic Stroke	17	1.74	79	1.55	1.10 (0.65–1.87)

HR: hazard ratio, CI: confidence interval; *p<0.05; **p<0.01; ***p<0.001.

The interaction *p* between age and septicaemia was <0.05 for all strokes, ischaemic and haemorrhagic stroke.

The interaction *p* between gender and septicaemia was 0.042 for haemorrhagic stroke but interaction *p* for all strokes and ischaemic stroke was >0.05.

**Figure 1:**
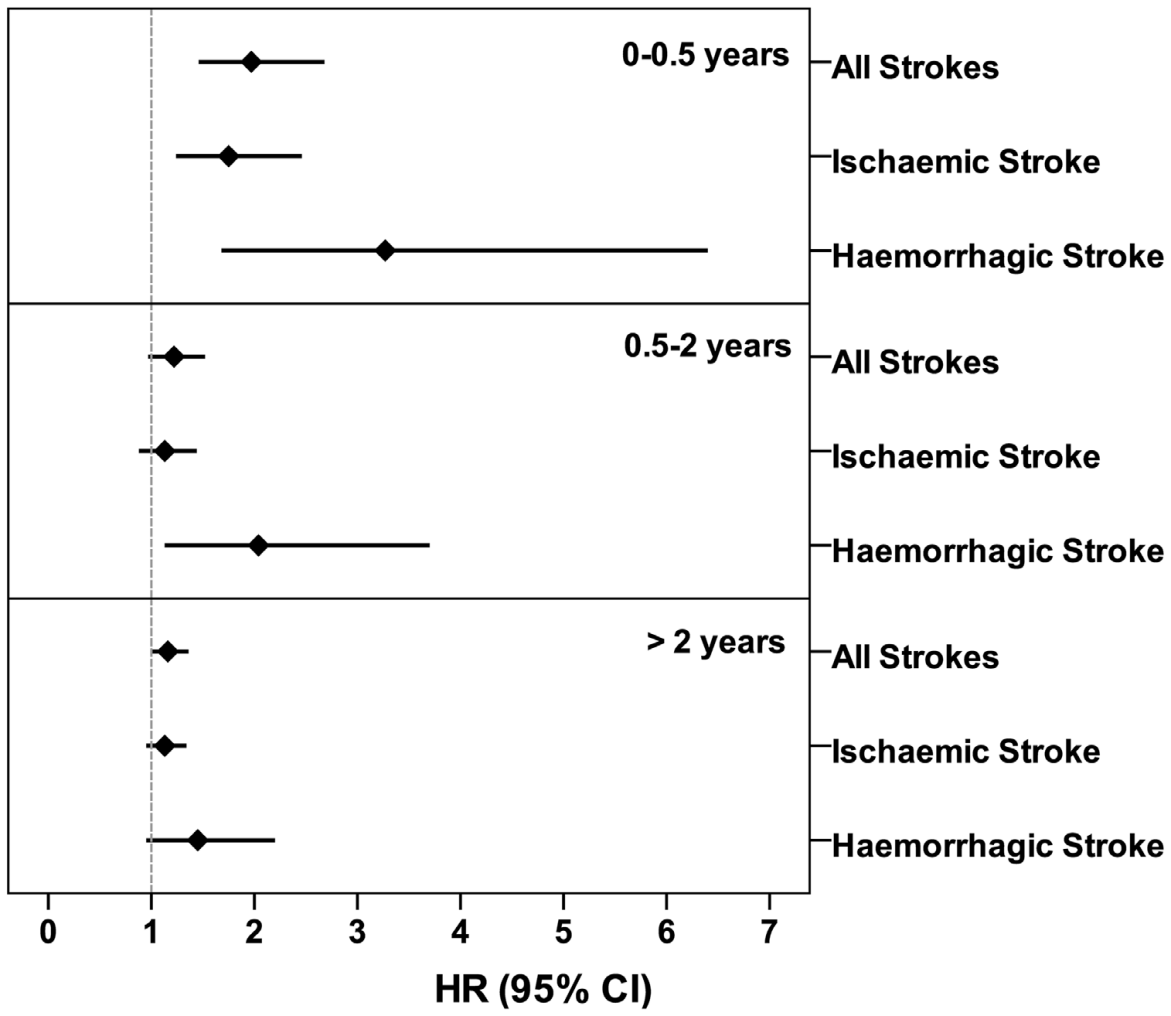
Age-, gender- and co-morbidities-adjusted hazard ratios for development of stroke following septicaemia at various time intervals.

Figure 1 shows the impact of time on the risk of developing stroke. A time-dependent decline was noted. The stroke risk was highest within the first 6 months of the septicaemia episode (stroke of any type, HR = 1.97, 95% CI: 1.46–2.68, *p*<0.001; ischaemic stroke, HR = 1.75, 95% CI: 1.24–2.46, *p* = 0.001; haemorrhagic stroke, HR = 3.27, 95% CI: 1.68–6.40, *p*<0.001). Beyond 6 months, there was a significant increase in stroke risk for haemorrhagic stroke within 2 years of the septicaemia episode (HR = 2.04, 95% CI: 1.13–3.70, *p*<0.05). Beyond 2 years (up to 10 years), there was no significant difference in overall stroke risk between the septicaemia group and the control cohort. The decline in stroke risk over time was significant (*p*<0.05) based on the relationship between Schoenfeld residuals for septicaemia and follow-up time in the Cox’s proportional hazard model.

## Discussion

This population-based longitudinal study was performed to assess whether septicaemia, an acute systemic infection, was associated with an increased risk for developing stroke. We showed that patients with septicaemia had a higher risk for developing ischaemic or haemorrhagic stroke. Comparison of the effect estimates (HRs) of stroke between the septicaemia and control groups showed significantly higher HRs in patients without co-morbidities compared with those with co-morbidities (established stroke risk factors including hypertension, diabetes, ischaemic heart diseases, atrial fibrillation and dyslipidaemia). It is worth noting that the risk for developing haemorrhagic stroke was higher than that for ischaemic stroke.

Septicaemia is a serious systemic infection characterized by intense inflammation intertwined with profound alteration of vascular function, often followed by a state of relative immune paralysis [17]. Although case-control studies suggested that recent infection (within 1 week) may be a trigger of stroke [9]–[12], results from the present study showed that septicaemia may have a sustained impact in increasing stroke risk especially in the first 6 months after septicaemia.

We showed that the young age group (<45 years) was more vulnerable to developing stroke compared to the middle aged group (45–64 years) which in turn was more vulnerable than the elderly (≥65 years) group. This was especially true for the haemorrhagic type that patients with septicaemia in the young age group had 28.6 times of risk for developing stroke compared to the control cohort, which was extremely higher to that of other types. Because of smaller case numbers in the young age group and in the group without co-morbidities, wider confidence intervals were noted. Male patients with septicaemia showed an increased risk of haemorrhagic stroke, around 2.1 times compared to the control cohort. The impact of septicaemia on stroke risk was greatest within 6 months of the septicaemia episode, and then declined gradually over time. These findings suggested that septicaemia increased the risk of developing stroke, especially the haemorrhagic type. We showed that the impact of septicaemia on stroke risk was more prominent in the younger population. This was consistent with studies showing that age is the most important risk factor for stroke [18]–[21]. Our data suggested that the greater effect size seen with septicaemia in younger patients could be due to the lower prevalence of established stroke risk factors in this group.

Inflammation and infection are gaining awareness and interest as potential conditions that increase stroke risk [22]. Systemic infections with resultant immune/inflammatory processes are related to stroke aetiology and pathology [23].

Chronic inflammation may influence the formation of atherosclerotic plaque [13], [24] and lipid metabolism, which are known to play a role in atherothrombotic diseases including stroke [25]–[27], and chronic inflammation has been added to the list of established risk factors for stroke [13].

Prior acute infections with *Mycoplasma pneumoniae, Helicobacter pylori, Chlamydia pneumoniae*, herpes virus, and cytomegalovirus are thought to contribute to the pathogenesis of ischaemic stroke, possibly resulting from the infectious burden, aberrant activation of the coagulation process, and endothelial dysfunction [28]–[32]. Systemic respiratory tract infections were shown to significantly increase the risk of stroke and myocardial infarction, especially during the first three days, supporting the notion that acute infections result in a transient increase in risk of vascular events [7]. These results were consistent with another study showing that individuals hospitalised for infections had an increased risk for stroke, with a higher risk within a short interval after infection [8]. Acute infections have been reported to increase plasma fibrinogen and CRP levels, which may affect the risk of stroke [33]. Acute respiratory tract and urinary tract infections have also been reported to independently increase the risk for ischaemic stroke [28]. The neuropathology of septic shock in a report on 23 patients who had died of this condition included cerebral haemorrhage, hypercoagulability syndrome, micro-abscesses, multifocal necrotising leukoencephalopathy, and ischaemia [34]. These studies suggest that identification of treatable infectious diseases associated with stroke risk may lead to more effective stroke prevention [28].

Based on these studies and our present results, we hypothesise that septicaemia may initiate an intense systemic inflammatory reaction and disordered coagulation process, resulting in structural changes in the cerebral vasculature, thereby increasing the risk for developing stroke. Septicaemia-induced structural changes in cerebral blood vessels may accelerate the pathogenic processes that culminate in earlier onset of stroke. The observation that septicaemia is associated with a higher risk for developing haemorrhagic stroke is also consistent with the contention that structural vascular changes associated with septicaemia could be the culprit. It will be interesting to further explore the pathogenic mechanisms underlying septicaemia-associated increase in stroke risk especially the haemorrhagic type.

An important strength of this study was the use of a population-based data set that allowed for inclusion of a large number of septicaemia patients, a sizeable number of whom eventually developed stroke during the follow-up period. We were able to obtain a well-matched control group without septicaemia from the same population for a long follow-up period of 7–10 years. The large sample sizes allowed subgroup analysis, which showed 1) a greater risk for developing haemorrhagic than ischaemic stroke following septicaemia, 2) a trend for the younger age group to be more vulnerable than the older group and 3) a significant decline in stroke risk over time.

### Limitations

This study has several limitations. First, data used in the present study did not include medications or lifestyle factors such as smoking, alcohol consumption, exercise habits and diet that may influence the risk of stroke. Second, data from a large dataset of this type may also include unidentified recurrent patients who may have had septicaemia and stroke before 1996 when NIH was started. Future prospective studies of patients with septicaemia will be necessary to expand and confirm our present results. The caveat associated with smaller case numbers in selected groups (young age groups, the groups without co-morbidities) should also be recognised. Finally, results presented in this study show only an association without establishing causality.

### Conclusion

In conclusion, to our knowledge, this is the first report to explore a possible association between septicaemia and stroke in a large nationwide population-based study. Septicaemia was associated with an increased risk of developing stroke, especially haemorrhagic stroke, particularly within the first 6 months of the septicaemia episode, with declining impact over time. Young patients (age <45 years) with septicaemia were more vulnerable to developing stroke, especially the haemorrhagic type, than the older group. Patients with septicaemia, who did not have the conventional stroke risk factors (co-morbidities) had a slightly higher risk for developing stroke, in particular the haemorrhagic type, than the control cohort. These findings suggest that stroke preventive measures could be improved by paying closer attention to subgroups of patients, particularly patients <45 years old who have no co-morbidities, within 6 months of a septicaemia episode.
